# On Restoration to Life

**Published:** 1848

**Authors:** Robert Brandon

**Affiliations:** London


### ARTICLE V.

(*When is a person dead ?*)—*on Restoration to Life after the Cessation of the Vital Functions.* By Robert Brandon, Esq., M.R.C.S., &c., London.

The London Lancet has received for publication, a paper under the above title, and gives the following analysis of it :

" Mr. Brandon starts by assuming that the electric and nervous fluids are one and the same ; nor does he believe that in cases which are to all appearance hopeless we should despair, since the co-application of transfusion and electricity will often achieve what the one or the other singly could not have effected. He then continues :

"'If decomposition be death, then every state short of decomposition must be either a condition between life and death, or life. It is to this state between life and death, or decomposition, that I apply the name latent; and I say life is latent, and can be recalled when latent, by transfusion and electricity, at any time previous to decomposition, provided the organs necessary for life are sufficiently sound to carry on the vital actions. And since by mere inspection we cannot tell the body which can, and the one which cannot be resuscitated, I think we should, in all cases of suspended animation, from accident or from disease, try to bring back the actions of vital organs, and thus place the body in the best condition we can for its recovery; and we can hope for great success in a majority of cases thought to be beyond the reach of surgery, on the account of the duality of organs and the increased activity in the functions of one-half of an organ when the other half is no longer capable of carrying on its actions.

"'If we can give blood to an animal which wants blood, as in approaching death, by means of transfusion—if we can give nervous power by electricity, which is considered to be identical with the vis nervosa—if we can give respiration to animals or men by means of artificial respiration and heat, which we can procure by artificial means, and through the agency of the established respiratory functions; I say, if we can do all these things in men and animals, we can place a moribund living creature in the situation to carry on the machinery of life; and in doing so, we call often into play the vital spark, the soul and the system of organic life.

"'Life is the *ensemble* of the functions called vital—viz.: respiration, circulation, &c.; animal heat is evolved when life is present. It is not necessary for me to write on the various theories of animal heat; the absence of heat, of the heart's action, and respiration, do not constitute death—for this only is present when putrefaction exist. If decomposition be the only true sign of death, then is every state prior to this, not death, but a condition intermediate between life and death. Life is then impassive, and persons may be, and often are, recovered from this state, which I call a state intermediate between life and death. Should this state be allowed to continue, and nature not effect a reaction, true death, or decomposition, ensues.

"'Look at a piece of sealing-wax; you see no evidence of the presence of electricity, yet is this present, and friction will at once make it evident.

"'The following declaration may startle many, and the idea will doubtless be repudiated, yet as the conviction is strong on my own mind, I will here declare my belief, that every one may be recovered, prior to decomposition, for a period, or entirely, according to the state of the organs necessary for life, and this recovery may be effected by means of galvanism, to give motion to the heart and other muscles: transfusion to give circulation and nourishment; the hot bath at 110°; friction, and the external and internal use of stimulants, as adjuncts to the treatment. The medical man's duty ends not with the cessation of the heart's action, as many are recovered from this state; nor is man dead or incapable of vitality until decomposition is present, and the advent of this must be prevented by the restoration of vitality. Houses for the reception of the dead, such as those at Frankfort, are much wanted in England, and in all cases, proper means should be used to discover those who may be recoved by the use of the treatment before indicated. Thus, premature burials will be prevented; and many a valuable life saved to society by science, may, perhaps, be the cause of the restoration of others from a state of simulated death.'"

